# Supplementary material for: Effect of Silver Modification on the Photoactivity of Titania Coatings with Different Pore Structures
Source: Nanomaterials (Basel). 2021 Aug 30;11(9):2240. doi: 10.3390/nano11092240 (PMC8466528; doi:10.3390/nano11092240)
Supplement: Supplementary file 1 [file nanomaterials-11-02240-s001.zip › nanomaterials-1356434-supplementary.pdf]

nanomaterials

MDPI

Supporting information

Effect of Silver Modification on the Photoactivity of Titania Coatings with Different Pore Structures

Borbála Tegze [1], Emőke Albert [1], Boglárka Dikó [1], Norbert Nagy [2], Adél Rácz [2], György Sáfrán [2], Attila Sulyok [2] and Zoltán Hórvölgyi [1,*]

S.1. XPS measurements

Table S1. Composition observed by X-ray photoelectron spectroscopy (XPS) measurements. Surface values show the composition at the very surface calculated using a homogeneous distribution model. Bulk values refer to the quantities determined by XPS after removing the surface atomic layers by ion sputtering.

		Na (at%)	O (at%)	Ti (at%)	Ag (at%)	Si (at%)
p-TiO_2	surface	2.1	74.3	23.2	-	0.4
	bulk	0.7	72.4	25.4	-	1.4
p-TiO_2(0.03Ag)	surface	5.2	72.8	18.4	0.1	3.5
	bulk	3.4	69.4	23.5	0.1	3.0
p-TiO_2(1Ag)	surface	2.1	71.6	23.5	0.3	2.5
	bulk	0.8	72.3	24.8	0.3	1.7
p-TiO_2-Ag	surface	2.4	74.5	20.2	1.2	1.7
	bulk	1.9	70.5	24.9	1.5	1.1
c-TiO_2	surface	9.8	65.3	20.9	-	4.0
	bulk	3.3	70.4	24.0	-	2.3
c-TiO_2(0.03Ag)	surface	3.4	70.8	23.0	0.2	2.7
	bulk	1.6	72.1	24.7	0.1	1.4
c-TiO_2(1Ag)	surface	2.6	70.4	23.8	0.8	2.4
	bulk	1.1	72.8	24.2	0.8	2.0

S.2. Wettability measurements

Table S2. Advancing (Θ_A) and receding (Θ_R) contact angles, Young's contact angle (Θ_{Young}) and contact angle hysteresis (H) measured on TiO_2 and TiO_2-Ag composite coatings.

Sample		Θ_A (°)	Θ_R (°)	Θ_{Young} (°)	H (°)
c-TiO_2	initial	28 ± 6	16 ± 2	23 ± 4	12 ± 5
	10 min UV	0	0	0	0
	30 min Vis	11 ± 11	1 ± 2	13 ± 10	10 ± 7
	1 day dark	43 ± 3	22 ± 2	34 ± 3	21 ± 2
	10 min UV	0	0	0	0
	30 min Vis	45 ± 4	25 ± 4	36 ± 4	20 ± 2
c-TiO_2(0.03Ag)	initial	30 ± 2	19 ± 6	25 ± 3	11 ± 4
	10 min UV	3 ± 4	0	2 ± 3	3 ± 4
	30 min Vis	26 ± 9	12 ± 7	20 ± 7	14 ± 3
	1 day dark	42 ± 4	21 ± 3	33 ± 3	21 ± 2
	10 min UV	0	0	0	0
	30 min Vis	63 ± 13	37 ± 9	51 ± 11	26 ± 4
c-TiO_2(1Ag)	initial	36 ± 3	24 ± 3	30 ± 1	12 ± 6
	10 min UV	4 ± 4	0	3 ± 3	4 ± 4
	30 min Vis	26 ± 8	11 ± 4	20 ± 6	15 ± 4
	1 day dark	44 ± 5	23 ± 4	35 ± 4	21 ± 1
	10 min UV	0	0	0	0
	30 min Vis	49 ± 4	27 ± 3	40 ± 3	22 ± 2
p-TiO_2	initial	16 ± 1	0	11 ± 1	16 ± 1
	10 min UV	0	0	0	0
	30 min Vis	19 ± 15	9 ± 8	15 ± 12	10 ± 8
	1 day dark	35 ± 4	17 ± 3	27 ± 4	21 ± 5
	10 min UV	5 ± 8	0	4 ± 6	5 ± 8
	30 min Vis	15 ± 24	9 ± 14	13 ± 19	7 ± 10
p-TiO_2(0.03Ag)	initial	15 ± 5	0	10 ± 3	15 ± 5
	10 min UV	3 ± 3	0	2 ± 2	3 ± 3
	30 min Vis	25 ± 9	11 ± 8	19 ± 8	14 ± 3
	1 day dark	31 ± 4	17 ± 2	25 ± 3	14 ± 4
	10 min UV	9 ± 7	0	7 ± 4	10 ± 6
	30 min Vis	35 ± 14	12 ± 13	27 ± 13	21 ± 3
p-TiO_2(1Ag)	initial	11 ± 10	0	8 ± 7	11 ± 10
	10 min UV	7 ± 1	0	5 ± 1	7 ± 1
	30 min Vis	25 ± 3	11 ± 2	19 ± 2	14 ± 3
	1 day dark	37 ± 4	19 ± 2	29 ± 4	20 ± 4
	10 min UV	4 ± 7	0	4 ± 5	6 ± 7
	30 min Vis	26 ± 9	13 ± 8	20 ± 9	15 ± 3
p-TiO_2-Ag	initial	21 ± 3	7 ± 2	16 ± 2	14 ± 2
	10 min UV	12 ± 9	0	8 ± 6	12 ± 9
	30 min Vis	50 ± 6	28 ± 3	40 ± 5	22 ± 4
	1 day dark	49 ± 2	26 ± 2	39 ± 2	23 ± 1
	10 min UV	16 ± 13	6 ± 5	12 ± 9	10 ± 8
	30 min Vis	56 ± 7	30 ± 4	45 ± 5	26 ± 4

S.3. Degradation rate constants

Table S3. Degradation rate constants (k_{m1}, k_{m2}) of rhodamine 6G and methylene blue monomer photodegradation under visible and UV irradiation, determined by the Julson-Ollis model.

irradiation	parameters	$c\text{-}TiO_2$	$c\text{-}TiO_2(0.03Ag)$	$c\text{-}TiO_2(1Ag)$
		Rhodamine 6G		
Vis	k_{m1} [min^{-1}]	0.032 ± 0.003	0.038 ± 0.007	0.048 ± 0.007
	k_{m2} [min^{-1}]	0.0019 ± 0.0001	0.0031 ± 0.0002	0.0032 ± 0.0003
UV	k_{m1} [min^{-1}]	0.1709 ± 0.0004	0.35 ± 0.02	0.254 ± 0.005
	k_{m2} [min^{-1}]	0.0260 ± 0.0003	0.043 ± 0.004	0.036 ± 0.002
		Methylene blue		
UV	k_{m1} [min^{-1}]	0.042 ± 0.003	0.043 ± 0.002	0.037 ± 0.005
	k_{m2} [min^{-1}]	0.0026 ± 0.0001	0.0026 ± 0.0002	0.0020 ± 0.0002

irradiation	parameters	$p\text{-}TiO_2$	$p\text{-}TiO_2(0.03Ag)$	$p\text{-}TiO_2(1Ag)$	$p\text{-}TiO_2\text{-}Ag$
		Rhodamine 6G			
Vis	k_{m1} [min^{-1}]	0.07 ± 0.01	0.066 ± 0.008	0.047 ± 0.004	0.032 ± 0.001
	k_{m2} [min^{-1}]	0.0042 ± 0.0002	0.00494 ± 0.00004	0.0036 ± 0.0004	0.0023 ± 0.0006
UV	k_{m1} [min^{-1}]	0.26 ± 0.02	0.201 ± 0.001	0.21 ± 0.06	0.41 ± 0.08
	k_{m2} [min^{-1}]	0.051 ± 0.002	0.03305 ± 0.00003	0.03 ± 0.01	0.044 ± 0.003
		Methylene blue			
UV	k_{m1} [min^{-1}]	0.0527 ± 0.0007	0.0574 ± 0.0099	0.045 ± 0.004	0.025 ± 0.008
	k_{m2} [min^{-1}]	0.0047 ± 0.0001	0.0048 ± 0.0007	0.0045 ± 0.0003	0.0020 ± 0.0003

Table S4. Degradation rate constants of rhodamine 6G monomer (k_{m1}, k_{m2}) and dimer (k_{d1}, k_{d2}, k_{dt}) photodegradation under visible and UV irradiation, determined by pseudo-first order model.

irradiation	parameters	$c\text{-}TiO_2$	$c\text{-}TiO_2(0.03Ag)$	$c\text{-}TiO_2(1Ag)$	
**Vis**	k_{m1} [min^{-1}]	0.00745 ± 0.00069	0.00856 ± 0.00073	0.0106 ± 0.0019	
	k_{m2} [min^{-1}]	0.00270 ± 0.00009	0.00351 ± 0.00023	0.00323 ± 0.00019	
	k_{d1} [min^{-1}]	0.00396 ± 0.00027	0.00365 ± 0.00022	0.00513 ± 0.00122	
	k_{d2} [min^{-1}]	0.00259 ± 0.00019	0.00354 ± 0.00033	0.00302 ± 0.00036	
	k_{dt} [min^{-1}]	0.00295 ± 0.00004	0.00388 ± 0.00019	0.00356 ± 0.00032	
UV	k_{m1} [min^{-1}]	0.0635 ± 0.0005	0.112 ± 0.004	0.0876 ± 0.0021	
	k_{m2} [min^{-1}]	0.0372 ± 0.0004	0.0489 ± 0.0025	0.0448 ± 0.0035	
	k_{d1} [min^{-1}]	0.0325 ± 0.0009	0.0557 ± 0.0019	0.0429 ± 0.0033	
	k_{d2} [min^{-1}]	0.0378 ± 0.0008	0.0502 ± 0.0017	0.0458 ± 0.0027	
	k_{dt} [min^{-1}]	0.0374 ± 0.0015	0.0542 ± 0.0018	0.0457 ± 0.0003	
**irradiation**	**parameters**	**$p\text{-}TiO_2$**	**$p\text{-}TiO_2(0.03Ag)$**	**$p\text{-}TiO_2(1Ag)$**	**$p\text{-}TiO_2\text{-}Ag$**
Vis	k_{m1} [min^{-1}]	0.0125 ± 0.0013	0.0125 ± 0.0009	0.0103 ± 0.0004	0.0078 ± 0.0005
	k_{m2} [min^{-1}]	0.00404 ± 0.00031	0.00482 ± 0.00001	0.00390 ± 0.00037	0.00312 ± 0.00066
	k_{d1} [min^{-1}]	0.00489 ± 0.00172	0.00382 ± 0.00067	0.00325 ± 0.00024	0.00134 ± 0.00043
	k_{d2} [min^{-1}]	0.00431 ± 0.00044	0.00500 ± 0.00001	0.00381 ± 0.00027	0.00266 ± 0.00094
	k_{dt} [min^{-1}]	0.00474 ± 0.00024	0.00539 ± 0.00020	0.00361 ± 0.00030	0.00261 ± 0.00094
UV	k_{m1} [min^{-1}]	0.0939 ± 0.0071	0.0738 ± 0.0003	0.0739 ± 0.0182	0.117 ± 0.014
	k_{m2} [min^{-1}]	0.0568 ± 0.0018	0.0404 ± 0.0013	0.0417 ± 0.0093	0.0490 ± 0.0017
	k_{d1} [min^{-1}]	0.0449 ± 0.0064	0.0205 ± 0.0009	0.0181 ± 0.0160	0.0472 ± 0.0090
	k_{d2} [min^{-1}]	0.0595 ± 0.0018	0.0400 ± 0.0024	0.0410 ± 0.0125	0.0521 ± 0.0004
	k_{dt} [min^{-1}]	0.0565 ± 0.0021	0.036 ± 0.0016	0.0375 ± 0.0126	0.0510 ± 0.0034

S.4. Quantum efficiency and quantum yield values

Quantum efficiency and quantum yield values were determined for the initial interval of R6G monomer degradation, under both UV and visible light (see in Table S5). Quantum efficiency (QE) is defined as the ratio between the number of degraded molecules/cm^2 and the number of incident photons/cm^2, while quantum yield (QY) is the ratio between the number of degraded molecules/cm^2 and the number of absorbed photons/cm^2. The total number of R6G dye molecules present in the dye impregnated coatings were estimated from dye desorption measurements. 3-3 samples of the different coatings were measured, by immersing a coating in 30 mL of distilled water, waiting 24 h for the desorption of dye molecules (equilibrium was reached, and it can be assumed that only a negligible amount of the dye molecules remained in the pore structure), then determining the concentration of the resulting dye solutions using the Lambert-Beer equation. The number of degraded dye molecules/cm^2 was then calculated using this result, and the degradation rates measured in the photodegradation experiments, and the area of the coatings. The number of incident photons/cm^2 was determined from the measured intensity of the lamps (at suitable wavelength values for the UV and visible light measurements). The number of absorbed photons/cm^2 was then calculated from this value by determining the percentage of absorbed light from UV-Vis spectroscopy measurements of different dye impregnated TiO_2 and TiO_2-Ag coatings (for the visible light measurements), and of titania coatings on quartz glass substrate (for the UV measurements). The percentage of absorbed light at a certain wavelength was estimated from the transmittance spectra and corrected with the reflectance. A more detailed description of the above summarized calculation process can be read in our previous publication [1].

Table S5. Quantum efficiency and quantum yield values of R6G monomer degradation (first interval) under visible and UV light.

	**Quantum efficiency (%)**		**Quantum yield (%)**	
	**Vis**	**UV**	**Vis**	**UV**
p-TiO_2	0.00742	0.189	0.0371	3.09
p-TiO_2(0.03Ag)	0.00828	0.173	0.0403	2.82
p-TiO_2(1Ag)	0.00766	0.188	0.0333	3.07
p-TiO_2-Ag	0.00433	0.201	0.0199	3.27
c-TiO_2	0.00479	0.136	0.0243	2.22
c-TiO_2(0.03Ag)	0.00609	0.245	0.0294	4.00
c-TiO_2(1Ag)	0.00840	0.251	0.0429	4.08

References

[1] Tegze B., Albert E., Fodor B., et al. Photoinduced processes of adsorbed and associated dye molecules in mesoporous titania coatings. *Dyes and Pigments*, **2019**, 167: 109–119.
